# Cannabinoids for behavioral symptoms in severe dementia: Safety and feasibility in a long-term pilot observational study in nineteen patients

**DOI:** 10.3389/fnagi.2022.957665

**Published:** 2022-09-29

**Authors:** Sophie Pautex, Federica Bianchi, Youssef Daali, Marc Augsburger, Christian de Saussure, James Wampfler, François Curtin, Jules Desmeules, Barbara Broers

**Affiliations:** ^1^Palliative Medicine Division, Department of Rehabilitation and Geriatrics, Geneva University Hospitals, Geneva, Switzerland; ^2^Faculty of Medicine, University of Geneva, Geneva, Switzerland; ^3^Long-Term Care Home “les Tilleuls”, Fondation pour l’Accueil et l’Hébergement de Personnes Âgées, Geneva, Switzerland; ^4^Clinical Pharmacology and Toxicology Division, Department of Anaesthesiology, Pharmacology, Intensive Care and Emergency Medicine, Geneva University Hospitals, Geneva, Switzerland; ^5^Institute of Pharmaceutical Sciences of Western Switzerland (ISPSO), University of Geneva, Geneva, Switzerland; ^6^Unit of Forensic Toxicology and Chemistry, University Centre of Legal Medicine Lausanne-Geneva, Lausanne University Hospital, University of Lausanne, Lausanne, Switzerland; ^7^Geneva University Hospital, University of Geneva, Geneva, Switzerland; ^8^Primary Care Division, Geneva University Hospitals, Geneva, Switzerland

**Keywords:** cannabinoids, dementia, symptoms relief, long-term care, medical cannabis

## Abstract

**Context:**

The management of behavioral symptoms and rigidity in patients with dementia constitutes a significant challenge. Short-term studies suggest an interest in the use of medical cannabis, but long-term data are lacking.

**Objectives:**

The objective of this study was to investigate the feasibility and long-term safety of administering tetrahydrocannabinol/cannabidiol (THC/CBD) treatment as an additional drug to a poly medicated population with severe dementia, evaluate clinical improvements, and collect information on the pharmacokinetics of cannabinoids and possible drug–drug interactions.

**Methods:**

A prospective observational study of patients with severe dementia living in a long-term care home to whom the physicians had prescribed a medical cannabis treatment. Data were collected over 2 years. We assessed the changes in medical cannabis dosages, safety parameters, variations in neuropsychiatric problems, agitation, rigidity, the most invalidating daily activity, and disabling behavior trouble scores. We evaluated the pharmacokinetics of cannabinoids by measuring plasma levels and analyzing the enzymatic activity.

**Results:**

We assessed 19 patients (81.4 years—17 women and two men) receiving an average of 12.4 mg THC/24.8 mg CBD per day for up to 13 months, with no reported problems related to the treatment and limited adverse drug reactions. Clinical scores showed a marked improvement that was stable over time, deprescription of other medications, and care facilitated. The pharmacokinetic evaluation showed an expected slight reduction in the enzymatic activity of CYP1A2 and CYP2C19.

**Conclusion:**

A long-term THC/CBD (1:2) medication can be administered safely and with overall positive clinical improvement to poly medicated older adults with severe dementia and associated problems. The results must be confirmed in a randomized trial.

## Introduction

Population expansion and aging contribute to a rise in the prevalence of dementia (Irwin et al., 2018). In 2020, more than 55 million people lived with dementia worldwide (Alzheimer’s Disease International, 2021). The medical and social care of patients with dementia represents a significant human, social, and economic investment (Prince et al., 2014). Neuropsychiatric symptoms or behavioral and psychological symptoms of dementia (BPSD) affect up to 97% of patients at a certain point in their illness (Steinberg et al., 2008). They are generally the most pervasive and complex symptoms to manage for relatives and caregivers (Beeri et al., 2002). BPSD significantly diminishes the quality of life of patients, family members, and healthcare staff. Management of these symptoms consists of correcting somatic and psychiatric factors, if possible, by implementing non-drug interventions such as psychological, psychosocial, and behavioral therapies (Cloak and Al Khalili, 2021). Nevertheless, despite these various interventions, medications must often be introduced, with limited effectiveness and side effects (Sink et al., 2005; Corbett et al., 2012).

The medical use of cannabis is gaining interest for different symptoms and diseases, including dementia and BPSD. *In vitro* and *in vivo* research suggests that cannabinoids may have neuroprotective, (Karl et al., 2012; Maroon and Bost, 2018) antioxidant, (Aso and Ferrer, 2014) immunosuppressive (Martín-Moreno et al., 2011; Karl et al., 2012), and anti-inflammatory (Karl et al., 2012; Aso and Ferrer, 2014; Liu et al., 2015) properties. According to pre-clinical studies, they may also contribute to reducing the amyloid plaque formation (Aso and Ferrer, 2014; Liu et al., 2015) and the neurofibrillary degeneration (Aso and Ferrer, 2014; Liu et al., 2015). Some experimental and clinical studies highlight the safety of administering cannabinoids to dementia patients. In addition, there is preliminary evidence that cannabinoids have the potential to alleviate neuropsychiatric symptoms such as agitation, irritability, delusions, and apathy (Hillen et al., 2019; Bahji et al., 2020).

Still, to our knowledge, there is a lack of data on the long-term safety of medical cannabis in vulnerable populations and real-life data about the medical cannabis pharmacokinetics in older poly medicated adults and its potential impact on cytochromes (CYP) activity. *In vitro* and *in vivo* studies evidence that cannabinoids interact with the cytochromes P450 and might be implicated in the interference of other drugs’ metabolism (Stout and Cimino, 2014; Alsherbiny and Li, 2018).

A pilot phase of 2 months during which the first patients were observed suggested overall clinical improvement, excellent tolerance, and deprescription of other medications (Broers et al., 2019). Therefore, this study is the follow-up of the pilot phase, with more patients included. The primary objective of this study was to investigate the feasibility and the long-term safety of administering a cannabis medication containing a standardized quantity of tetrahydrocannabinol (THC) and cannabidiol (CBD) to patients with severe dementia and living in a nursing home. The secondary objective was to collect information on the clinical outcomes, the possibility of concomitant drug deprescription, the potential drug–drug interactions, and the therapeutic drug monitoring (TDM) of the cannabis treatment.

## Methods

This is a prospective observational study of 19 patients who received medical cannabis (off-label use) prescribed by their physician with special authorization from the Federal Office of Public Health (FOPH). Medical cannabis is available in Switzerland for specific indications such as chronic pain, spasticity, and spasms for individual patients following a medical prescription with special authorization.

The study protocol was approved by the Geneva Ethics Committee (protocol number 2017-01196), and formal informed consent was approved and signed by a close relative or family member.

### Setting

Participants lived in a long-term care home (”Résidence Les Tilleuls”) in Geneva that is specialized in caring for residents with severe dementia from different origins (Alzheimer’s disease, vascular, and mixed) (McKhann et al., 1984; Román et al., 1993). The permanent staff includes nurses, nurse aids, and an attending physician.

### Criteria for cannabinoid prescription and administration

The physician proposed medical cannabis to patients with advanced dementia, whose medical treatment was carefully analyzed and optimized but presented persisting behavioral disorders. The family had to give written consent. The treatment was not administered in the presence of any unstable somatic disease requiring frequent drug adaptations, severe heart failure, symptomatic orthostatic hypotension, antidepressant treatment with tricyclic drugs, serotonin reuptake inhibitor fluoxetine, and the antiepileptic carbamazepine.

### The medical cannabis treatment

The treatment was an oral standardized Cannabis Sativa tincture or oil extract with 10 mg THC/ml and 20 mg CBD/ml, purchased at a federally licensed pharmacy in Langnau (BE-CH) and never changed during the study observation period. The standardized cannabis extract is from domestic hemp cultivation authorized by the FOPH, 1 g of natural hemp extract contains approx., 11 mg THC, and approx. 22 mg CBD. Each individually produced batch is tested, and a corresponding certificate of analysis is available.

The alcoholic solution contains approx. 80% (V/V) ethanol. The base for the oil is an organic hemp oil stabilized with ascorbyl palmitate, a natural preservative. Cannabinol (CBN) is a degradation product of THC. The monograph «Cannabis flos, Cannabisblüten» of the Swiss Pharmacopeia (Pharm Helv) requires a maximum content of 1% in Cannabis flowers. All the results of sample analysis showed CBN contents <0.3 mg/g = <0.03%.

The medication was introduced following the principle “start low, go slow,” with a daily intake divided into three doses and optimized by individual titration. The doses were adapted according to the physician’s assessment evaluating the benefits and the possible adverse effects.

Nabiximols oromucosal spray, the only registered cannabis-based medication available, was not considered by the physician since previous experiences showed difficulty spraying the product correctly in patients’ mouths.

### Participants

The eligibility criteria were patients (>18 years old) with severe dementia of different origins (McKhann et al., 1984; Román et al., 1993) as diagnosed by the physicians, confirmed by a neuropsychological assessment at the admission in the facility, and a Mini-mental State Examination score (Perneczky et al., 2006) (MMSE) <10, consistent with advanced dementia. The legal representative had to consent since all patients were formerly assessed for mental incapacity. Patients were to be excluded in the absence of the signed consent or when showing signs of unwillingness to participate. The administration of off-label use of medical cannabis was authorized for all patients before inclusion.

### Safety assessments

The healthcare professionals recorded all the adverse events, severity, and management. In addition, the physician and the principal investigator evaluated the severity and the possible relatedness to the medical cannabis treatment. In addition, blood pressure and weight were assessed at each visit. The adverse events were evaluated in severity and causality following the criteria of clinical trials as listed in ICH E.6 (R2) and E2A guidelines (ICH, 2022) and the WHO–UMC causality assessment criteria (Uppsala Monitoring Centre, 2022).

### Clinical assessments

One trained nurse, working as a supervisor in the nursing home and not involved in the direct care of patients, performed the assessments the day before introducing the cannabis medication, after 2 weeks, 1, 2, 3 months, and then every 2 months.

The 24 h dosage of THC/CBD administered and concomitant medications were recorded at each visit. The scores assessed to evaluate the clinical improvement in BPSD were as follows: (1) The Cohen–Mansfield Agitation Inventory (Cohen-Mansfield and Deutsch, 1996) score to assess patients’ agitation and its frequency. (2) The Neuropsychiatric Inventory (NPI) score (Cummings et al., 1994) to evaluate the severity and frequency of 12 primary behavioral dementia-related problems. (3) The Unified Parkinson’s Disease Rating Scale item 22, (Richards et al., 1994) to evaluate patient rigidity with passive movements. (4) A visual analog scale determined by the staff, scoring 0 to 10 to determine the most invalidating daily activities—mainly hygiene care, toileting, and dressing. (5) A visual analog scale determined by staff, scoring 0 to 10, to determine the most invalidating behaviors—mainly opposition, shouting, vulgarity, moaning, and wandering.

### Evaluation of tetrahydrocannabinol/cannabidiol on cytochromes activity

The enzymatic activities of the 6 enzymes—CYP1A2, CYP2B6, CYP2C9, CYP2C19, CYP2D6, and CYP3A4/5—were assessed at two-time points about 6 months apart, after at least 1 month of cannabis intake, with stable dosages for at least 15 days. Dosages were sometimes incremented between the two samplings.

Participants were given a validated test [the Geneva phenotyping micro cocktail (Bosilkovska et al., 2016)] to evaluate the first step of monooxygenase metabolism, essentially CYP450 isoform activity, in the morning in a fasting condition. The metabolic ratio (metabolite concentration/substrate concentration) of each CYP-specific probe substrate was measured in plasma 2 h after micro cocktail intake. The analysis was conducted through liquid chromatography–tandem mass spectrometry LC-MS/MS (Bosilkovska et al., 2014).

### Pharmacokinetics of tetrahydrocannabinol/cannabidiol

Therapeutic drug monitoring of THC/CBD was measured at a steady state, 12 h after the last drug intake, characterizing the plasma through levels of THC, its two metabolites 11-hydroxy-tetrahydrocannabinol (11-OH-THC) and free carboxy-tetrahydrocannabinol (THC-COOH), and CBD. Concentrations were determined using LC-MS/MS (Fabritius et al., 2013).

### Collection of data and statistical analysis

Under the supervision of senior physicians, two medical students and a pharmacy student collected the data from the medical files and, during meetings with staff, anonymized and entered them in clinical record files in an electronic data capture software (REDCap). Descriptive statistical analyses were carried out using Excel^®^ (Microsoft) software. Min/max values, mean, standard deviations (SD), median, and dispersion around the median (Q1 and Q3 quartiles) were calculated.

The enzymatic activity of cytochromes was evaluated through the metabolic ratio values (metabolite concentration/substrate concentration), their dispersion from the median, and their comparison to Geneva cocktail standard reference values (Bosilkovska et al., 2016).

## Results

### Population, follow-up period

Twenty patients had the off-label authorization for the cannabis medication in the long-term home between December 2017 and July 2019. Nineteen participants, whose demographic and clinical characteristics are described in Table 1, were gradually prospectively included, and data were collected until February 2020. The population was composed of 17 female patients (89.5%) and two male patients (10.5%), reflecting the gender distribution of the patients in the facility. Patients were affected by sporadic dementia, had different comorbidities, and generally took several co-medications, with an average of seven drugs. The average follow-up was 10 months (min = 5; max = 13 months), and the period varied depending on the date of inclusion. For two patients, the family asked to stop the cannabis treatment after 5 months because they did not find it effective enough.

**TABLE 1:** Baseline characteristics.

**Residents (*n*)**		19
**Sex (*n*; %)**		
Male		2 (10.5%)
Female		17 (89.5%)
**Characteristics (Mean ± SD; Range)**		
Age, years		81.4 ± 7.7; 61–95
Weight, kg		61.7 ± 8.6; 50.5–76.5
Blood pressure, mmHg	Systolic	129.2 ± 17.5; 105.0–160.0
	Diastolic	70.0 ± 6.5; 60.0–80.0
MMS		1,4 ± 2.4; 0–9
**Diagnosis of dementia (*n*; %)**		
Alzheimer’s disease		11 (57.9%)
Vascular		4 (26.3%)
Parkinson’s disease		1 (5.3%)
Unspecified		2 (10.5%)
**Comorbidities (*n*; %)**		
Neurological system disease (of which epilepsy)		4 (26.3%) [3 (15.8%)]
Cardiovascular disease		3 (15.8%)
Gastrointestinal disease		2 (10.5%)
Urogenital system disease		2 (10.5%)
Psychiatric disease		1 (5.3%)
Respiratory system disease		1 (5.3%)
Others (including locomotor and endocrine system disease)		8 (42.1%)
**Concomitant medications (Mean ± SD; Range)**		
Psychotropics, analgesics, diuretics, laxatives, cardiovascular system treatments		7 ± 2; 3–11

### Medical cannabis administration feasibility and dosages over time

The first participants received a THC/CBD tincture diluted in water with syrup. After a few weeks of treatment, some patients presented mouth pain and gingivitis that disappeared after changing the tincture to a THC/CBD oil formulation. Finally, the greasy and bitter oil, dripped in a “fromage frais” or a fruit jelly, was entirely administered to patients without difficulty. After that, this oil formulation was adopted, resulting in patients’ good acceptance.

**FIGURE 1:**
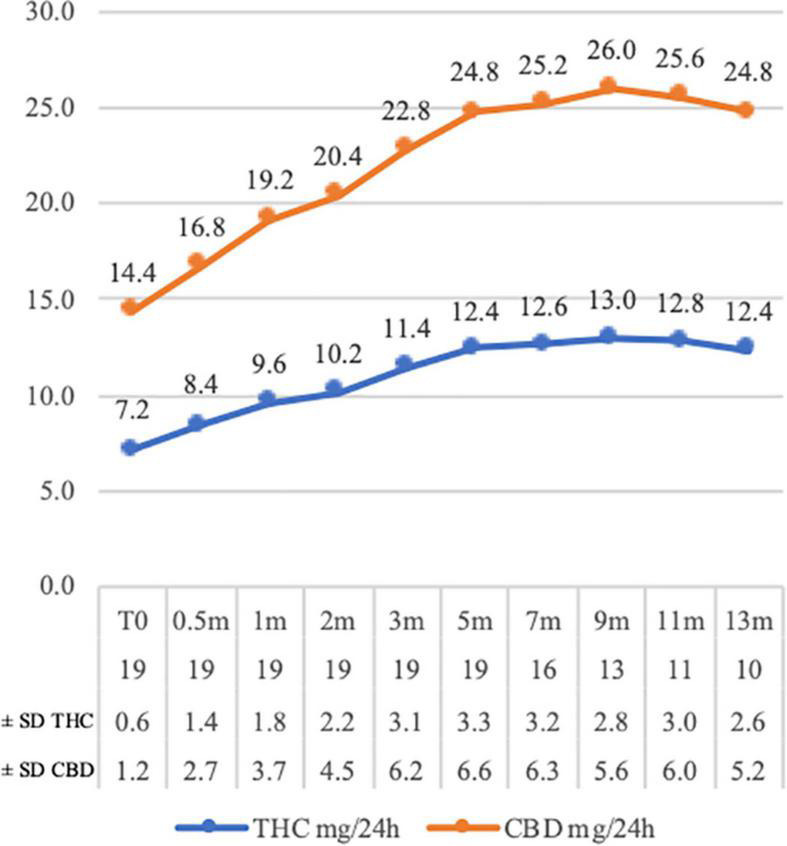
Tetrahydrocannabinol (THC) and cannabidiol (CBD) mg average daily dosage variations at the time of assessments (CBD dosages are double THC dosages). The number of assessed patients and the dosage standard deviations (THC and CBD) are proposed in the chart legend at each time point. *m* = month of assessment.

The evolution of the average dosage is described in Figure 1: The starting average dosage was 7.2 mg THC/14.4 mg CBD per day (3 times over the day), and the average dosage was 12.4 mg THC/24.8 mg CBD in 24 h after 13 months (± 2.6 mg THC/ ± 5.2 mg CBD; 9.6 mg THC/19.2 mgCBD-15.6 mgTHC/31.2 mg CBD) (The per-patient evolution of dosage changes in, Supplementary Figure A1).

### Safety-related events and adverse events

None of the patients had to stop the treatment for CBD or THC side-related effects. A total of 117 adverse events were recorded, of which only three serious adverse events (2.6%) were reported and were considered unrelated to the cannabis treatment by the physician and the principal investigator. They included two severe events: one hospitalization for high blood pressure in a patient refusing to take her antihypertensive drugs, and one hospitalization for entropion needing surgery. The third SAE was the death of a patient 8 months after inclusion in the study. There was no obvious cause for the death, but this occurred in a female patient suffering from long-duration cerebrovascular and circulatory disorders. The causal link with the intake of medical cannabis was not plausible.

According to the evaluation based on the healthcare professionals’ reports, 74 events (63.2%) were considered mild and resolved spontaneously, and 40 events (34.2%) were considered moderate and needed minor local interventions. Moreover, 11 events (9.4%) were considered definitely related to the cannabis treatment; of those, three were mouth pain, oral mucosal disorder, and mouth bleeding, resolved after the replacement of the alcoholic formulation, and six were eye disorders in one patient in the month before dying. Five events (4.3%) were probably related, and seven events (5.9%) were possibly related to the study treatment; of those, seven events (three probable and four possible) were mouth disorders that disappeared after changing to the oil formulation. For a detailed description, see Supplementary Table A1.

Systolic and diastolic blood pressure was stable, and participants’ mean weight decreased slightly, with significant variability and individual up and down fluctuation.

### Clinical evolution

**FIGURE 2:**
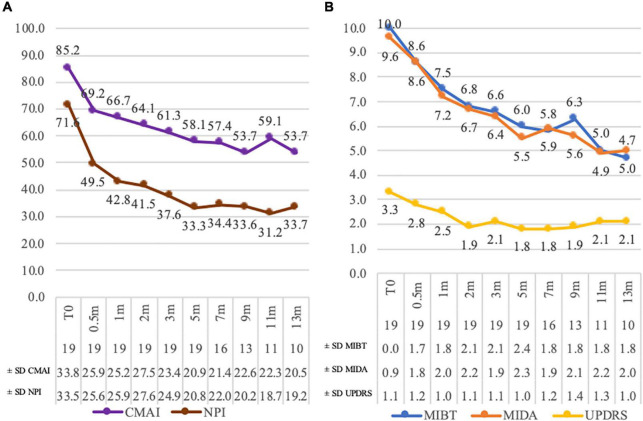
Assessment score variations. Panel (A): Cohen–Mansfield agitation score (CMAI) and neuropsychiatric inventory (NPI) scores. Panel (B): Unified Rating Scale for Parkinson’s disease item 22 (UPDRS), most incapacitating daily activity (MIDA), and most incapacitating behavioral trouble (MIBT) scores. The number of assessed patients and the score standard deviations are in the chart legend at each time point. *m* = month of assessment.

The Cohen-Mansfield agitation score (CMAI) average reduction was from 85.2 (± 33.7; 35–144) to 53.70 (± 20.5; 31–98) at 13 months (Figure 2A). The NPI decreased from 71.6 (± 36.5; 24–132) to 33.7 (± 19.2; 4–60) after 13 months (Figure 2A).

The average Unified Rating Scale for Parkinson’s disease item 22 (UPDRS) score reduced from 3.3 (± 1.1; 0–4; 79% of patients presenting a grade 4 or 3) to 2.1 (± 0.9; 0–3; 63% of patients showing a grade 2 or lower) at 13 months. Two patients (10%) had a decrease of three points and eight (42%) of two points. An unchanged score was registered for three residents (16%), of whom one had a zero score since the beginning (Figure 2B). The most frequent incapacitating daily activities (MIDA) were difficulty with personal daily care (84%) and dressing or undressing (37%). A reduction in the score means that the activity is done with less difficulty for patients. At 13 months, the average score decreased from 9.6 (± 0.9; 7–10) to 5.0 (± 2; 2–8) (Figure 2B). The average score for the most incapacitating behavioral trouble (MIBT) dropped from 10 (min = max = 10) to 4.7 (± 1.8; 2–7) at 13 months. The most frequent troubles were screaming (39%) and opposition (58%) (Figure 2B).

A deprescribing of several drugs occurred following the introduction of medical cannabis. The classes most concerned were central analgesics (morphine type), typical antipsychotics (haloperidol and levomepromazine), atypical antipsychotics (quetiapine and risperidone), and antidepressants (trazodone and escitalopram). The interruption of four psychotropic drugs was possible for two patients (10%) without problem emerging. One drug prescription was discontinued in 14 patients (84%), and of those, five patients (23%) needed the re-introduction of one psychotropic drug. No drug changes were recorded for three patients (16%). For a detailed description, see Supplementary Tables A2, A3.

### Phenotyping of cytochromes P450

The evaluation of the enzymatic activity was conducted on 15 patients. For three patients, a concomitant medication was stopped between the two samplings: one discontinued risperidone, one haloperidol, and one pregabalin.

**FIGURE 3:**
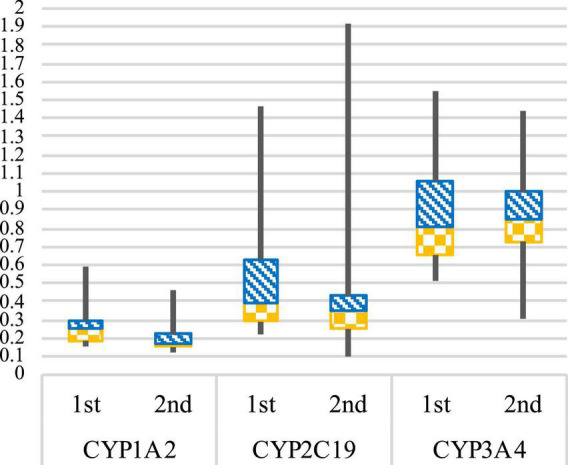
CYP1A2, CYP2C19, and CYP3A4 enzymatic activities. Box plot representing the CYP1A2, CYP2C19, and CYP3A4 metabolic ratios at 1st and 2nd blood sampling. Geneva cocktail reference values (–SD to + SD): CYP1A2 = PM (poor metabolizer) 0.03–0.204; EM (normal metabolizer) 0.17–0.39; UM (ultra-metabolizer) 0.42–0.70. CYP2C19 = PM (poor metabolizer) 0.08–0.28; EM (normal metabolizer) 0.3–1.22; UM (ultra-metabolizer) 2.96–7.88. CYP3A4 = PM (poor metabolizer) 0.15–0.29; EM (normal metabolizer) 0.32–0.82; UM (ultra-metabolizer) 2.21–5.25. For CYP2C9, CYP2B6, and CYP2D6 graphics in Supplementary Figures B1, B2.

The evaluation of the enzymatic activity of cytochromes by phenotyping, in Figure 3, showed a slowdown in the enzymatic activity of the CYP1A2 and CYP2C19 with a phenotyping switch between the two blood samplings (Metabolic ratios mean ± SD: CYP1A2 1st sampling = 0.26 ± 0.11, 2nd sampling = 0.20 ± 0.09; CYP2C19 1st sampling = 0.55 ± 0.38, 2nd sampling = 0.44 ± 0.44). At the first sampling, five patients (33%) were categorized as slow CYP1A2 metabolizers (PM). However, ten patients (67%) resulted in slow metabolizers at the second sampling.

For CYP2C19, three patients (20%) were categorized as slow metabolizers at the first sampling and five (33%) at the second. No concomitant drugs could explain the results observed, apart from one patient taking esomeprazole, a strong CYP2C19 inhibitor, at both samplings.

For CYP3A4, the enzymatic activity remained stable between the two blood samplings, and no other phenotyping switches were observed (Metabolic ratios mean ± SD: CYP3A4 1st sampling = 0.89 ± 0.32, 2nd sampling = 0.85 ± 0.27).

A full report of values and graphics is in Supplementary Tables B1, B2 and Supplementary Figures B1, B2.

### Determination of plasma levels of cannabinoids

Results are available for 18 patients (total values report in Supplementary Table B3). Tetrahydrocannabinol plasma concentrations below 1 μg/L or not detectable represented 72% of all samples (mean = 1.4 ± 0.9; ≤ 1–4.7 μg/L). 11-OH-THC plasma concentrations were comparable to the THC (mean = 1.9 ± 1.7; ≤ 1–8.9 μg/L). THC-COOH plasma concentrations mean value was 54.0 μg/L (±28.1; 15–106 μg/L). The treatment had been stopped for two patients for 1 and 4 months at the second sampling, and for them, all plasma concentrations were not detectable.

## Discussion

Our study offers new perspectives on the possibility of improving the quality of care for patients with severe dementia. A THC/CBD (1:2) medication was administered, and we observed the patients for 13 months, the most extended study observation period so far reported to our knowledge. In the few studies on cannabinoids for dementia, the length of treatment was always short and varied from 3 days/week of administration for 6 weeks (Ahmed et al., 2015; van den Elsen et al., 2015) to a maximum of 6 weeks of continuous treatment (Volicer et al., 1997). We also observed higher dosage prescription of THC compared to other studies where they ranged from 1.5 mg THC (Amanullah et al., 2013; van den Elsen et al., 2015) per day to a maximum of 14 mg THC in one study (Shelef et al., 2016). We observed the slow increase of the dosages to a mean maximum of 14.3 mg THC with an average intake at 13 months of 12.4 mg daily.

A synthetic THC analog was used in most studies apart from one (Shelef et al., 2016) in which the study treatment was a medical cannabis oil containing 1.65% of THC and less than 0.05% of CBD. In addition, the use of combined therapy of THC and CBD was reported in studies on other indications [e.g., chronic pain (Überall, 2020)], and the use of CBD alone is noted for the treatment of psychosis in Parkinson’s disease (Zuardi et al., 2009). The patients of this study received a natural plant formulation standardized in THC and CBD (1:2) contents according to the hypothesis that the natural extract might have better tolerability and efficacy, the presence of CBD counterbalancing the psychoactive effects of THC (Niesink and van Laar, 2013; MacCallum and Russo, 2018).

The practical administration of medical cannabis was possible after some adaptations, and we observed an improvement in behavioral troubles and rigidity. We observed the most marked improvements in the weeks after introducing the cannabis medication; then, they stabilized and lasted throughout the observation period. This led to better daily care for health professionals, greater acceptance of care assistance and easier implementation of non-drug interventions, and potentially reduced caregiver burden (Beeri et al., 2002; Corbett et al., 2012). Still, in two cases, the families asked to stop the cannabis medication after 5 months, even though there were no drug-related adverse events since they considered there was not enough improvement. We observed that the deprescription of other drugs, especially neuroleptics, was possible after the cannabinoid introduction. Nevertheless, psychotropic medications had to be reintroduced in some cases.

Patients tolerated well the dosage titration adjustments with a favorable safety profile. The mean plasma THC-COOH concentrations around 55 μg/L or higher are the dosages expected for habitual THC use and show that the treatment was regularly taken and well absorbed (Giroud et al., 2001; Fabritius et al., 2014). Still, no severe adverse events related to the treatment were reported, and no drug discontinuation was observed due to side effects. Concerning drug interactions in a vulnerable, multi-medicated population, the blood sampling and the phenotyping conducted within 6 months of interval did not show an accumulation of THC or its metabolites nor of CBD over time. Furthermore, the two patients who interrupted the drug intake did not present any THC, CBD, or metabolites residual plasma concentrations.

Nevertheless, a slight reduction in some hepatic enzymatic activity was observed. Notably, CYP1A2 and CYP2C19 showed a phenotyping switch in some patients by a lowering in their activity that must be considered in the presence of co-medications metabolized and eliminated on the same enzymatic route. The blockade of CYP1A2 might be important for some drugs, such as antidepressants (e.g., fluvoxamine and duloxetine), neuroleptics (e.g., clozapine, olanzapine, and quetiapine), and myorelaxants (e.g., tizanidine), which might accumulate if the dosage is not adapted. Furthermore, the blockade of CYP2C19 might interfere with the metabolism of some antidepressants (e.g., citalopram, amitriptyline, clomipramine, and fluoxetine) and psychotropic drugs (e.g., galantamine). Also, cardiovascular drugs like clopidogrel necessitate CYP2C19 to bioactivate, and it could remain inefficient in the presence of concomitant medications interfering with the hepatic enzymatic activity. However, only these two cytochromes were impacted, and we did not see any other changes in CYP or side effects that could have been caused by this slowing down of the enzymatic activity or the accumulation of other medications.

Inhibition of the CYP3A4 activity is suggested in the SmPC for nabiximols at high dosages or over time (Sativex, 2021). However, the CYP3A4 enzymatic activity was almost unchanged in our cohort even despite a long period of medication intake. The THC/CBD ratio, as well as the administration route and the formulation, might change the metabolic pathway of the cannabinoids, thus influencing drug interactions (Alsherbiny and Li, 2018). Moreover, the generally weak influence of the cannabis treatment on the cytochromes could be partly explained by the low plasma concentrations observed in all patients. Still, we cannot exclude the effect of being higher by increasing the THC/CBD dose.

Limits of this study include the uncontrolled observational design, so caution is needed to interpret the data and causality between the medication intake and the encouraging results. Also, the gender distribution limits the interpretation, and the sex-related differences might influence cannabinoid activity (Fattore and Fratta, 2010). In addition, the study nurse was aware of the treatment, which might represent a bias in the scores assessments. Furthermore, the cannabis treatment dosage adjustments were not pre-defined but were decided by the physician based on individual observation. Also, the concomitant drug deprescription was not pre-determined but proposed considering the marked improvement in behavioral troubles and rigidity.

Relatedness between the deprescription of psychotropic drugs because of the slowing down of the enzymatic activity of CYP1A2 and CYP2C19 cannot be excluded. Therefore, only a complete pharmacological analysis should confront the data collected to values taken before introducing medical cannabis. In addition, the genotype information might help determine the predictive activity of each CYP and help discriminate the influence of the environment since no change in the activity is expected when a patient is a genetically poor metabolizer, even if an inhibitor is given.

Despite the limits of this study, the positive overall results on rigidity, care, and behavior in this population are encouraging. Also, the pharmacokinetic profile is promising. Moreover, the sociological perspective of medical cannabis prescription was highly favorable. The caregivers, initially showing reluctance, particularly appreciated the improved quality of the contact with their patients. Furthermore, families were enthusiastic about offering their relatives an alternative and accepted the treatment without concern (Revol, 2019).

Those preliminary data can help future researchers to set up clinical trials to prove the efficacy of medical cannabis. Indeed, based on these favorable preliminary results, we are currently pursuing our investigation by setting up a randomized clinical trial to show the efficacy and safety of cannabis oil for BPSD.

## Conclusion

To our knowledge, this is the first study of this kind, with the administration of medical cannabis for an extended period in a highly vulnerable poly medicated demented population conducted within a “natural” setting. The natural cannabis oil has a THC: CBD proportion of 1:2, where CBD possibly reduces the THC psychoactive properties and other side effects previously reported with synthetic THC formulations. The dosages used in this study were significantly higher than the ones reported in other studies, even if the safety limits proposed for other pathologies were respected. Despite the particularity of the population, in which we could expect adverse events or complications, the side effects were limited. Also, the pharmacological profile was favorable and did not provide evidence of critical drug–drug interactions. The interest in medical cannabis is rising. Based on the results of this study, this new approach might represent a valid, feasible, and safe alternative for patients with dementia.

## Data availability statement

The original contributions presented in this study are included in the article/Supplementary material, further inquiries can be directed to the corresponding author.

## Ethics statement

The studies involving human participants were reviewed and approved by the Commission Cantonale d’Éthique de la Recherche Genève. The patients/participants provided their written informed consent to participate in this study.

## Author contributions

SP and BB designed the research protocol and supervised the study. Jointly with JD, YD, and MA they supervised the data collection and analysis. FC and FB analyzed the data, the latter providing this manuscript. CS and JW made the whole study possible, initiating the cannabinoid prescription, and providing all the practical assistance to the conduct of the study. All the authors conducted a critical revision of the manuscript.
