# Erratum: Cannabinoids for behavioral symptoms in severe dementia: Safety and feasibility in a long-term pilot observational study in nineteen patients

**DOI:** 10.3389/fnagi.2023.1176468

**Published:** 2023-03-09

**Authors:** Frontiers Production Office

**Affiliations:** Frontiers Media SA, Lausanne, Switzerland

**Keywords:** cannabinoids, dementia, symptoms relief, long-term care, medical cannabis

An omission to the funding section of the original article was made in error. The following sentence has been added: “Open access funding was provided by the University of Geneva.”

The original article has been updated.

