# Supplementary material for: Cannabinoids for behavioral symptoms in severe dementia: Safety and feasibility in a long-term pilot observational study in nineteen patients
Source: Front Aging Neurosci. 2022 Sep 29;14:957665. doi: 10.3389/fnagi.2022.957665 (PMC9557769; doi:10.3389/fnagi.2022.957665)
Supplement: Supplementary file 1 [file Data_Sheet_1.pdf]

Supplementary material

Supplement A

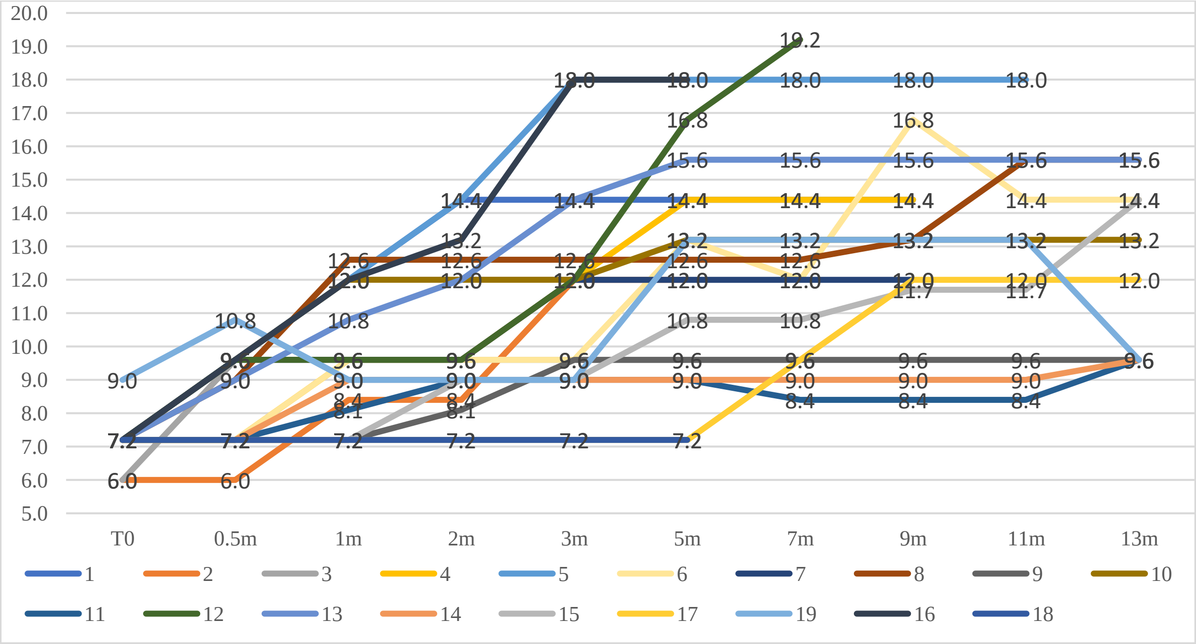

Figure A1: per-patient THC mg dosage evolution at time of assessments. m=month of assessment

Table A.1 – Adverse events

**Adverse event (MedDRA System Organ Class)**	**Number of events**	**Relatedness**	**Assessment of severity**
Blood and lymphatic system disorders	1	Not related	Moderate
Ear and labyrinth disorders	1	Not related	Mild
Endocrine disorders	1	Not related	Moderate
Eye disorders [a]	9	1 Definitely-1 Probable<br>6 Not related	7 Mild/1Moderate<br>1 Severe
Gastrointestinal disorders	26	1 Possibly-2 Unlikely<br>23 Not related	14 Mild /12 Moderate
General disorders [b] and administration site conditions [c]	12	3 Definitely-3 Probable<br>4 Possible -2 Not related	9Mild/2Moderate/1 Death
Infections and infestations	17	17 Not related	6Mild/11Moderate
Metabolism and nutrition disorders	9	1 Definitely-1 Probable<br>7 Not related	8Mild/1Moderate
Musculoskeletal and connective tissue disorders	6	6 Not related	5Mild/1Moderate
Neoplasms benign, malignant, and unspecified (including cysts and polyps)	2	2 Not related	1 Mild/1 Moderate
Nervous system disorders	14	1 Definitely-2 Possible<br>11 Not related	11Mild/3 Moderate
Psychiatric disorders	1	Unlikely	Moderate
Renal and urinary disorders	1	Not related	Mild
Respiratory, thoracic, and mediastinal disorders	6	2 Unlikely-4 Not related	1 Mild/5Moderate
Skin and subcutaneous tissue disorders	6	1 Probable -5 Not related	6 Mild
Vascular disorders	5	1Unlikely-4 Not related	4Mild/1Severe

a. Eye disorders were recorded for three patients: one had mild red eyes, one experimented eyelid hematoma and tearing eyes in the month before dying, one suffered from entropion and needed surgery

b. General disorder : Death (not related)

c. Administration site disorders : mouth pain, mouth ulcers, and gingivitis - were resolved when the treatment was changed from the alcoholic tincture to the oil formulation and never occurred after.

Table A.2

Deprescribed drugs and time at deprescription since first cannabinoids intake

Study Id	ATC treatment - deprescription	Time
2	risperidone	10 weeks
3	quetiapine	2 weeks
4	quetiapine	3 weeks
5	celecoxib	12 weeks
6	haloperidol	64 weeks
7	morphine	8 weeks
8	morphine	16 weeks
9	diclofenac	48 weeks
10	levodopa and decarboxylase inhibitor	2 weeks
	haloperidol	15 weeks
	morphine	48 weeks
	morphine*	3 weeks
12	pregabalin	15 weeks
13	pregabalin	4 weeks
14	trazodone	5 weeks
16	trazodone	22 weeks
17	quetiapine	76 weeks
18	escitalopram	26 weeks
19	haloperidol	17 weeks
	levomepromazine	17 weeks
	valproic acid	52 weeks
	morphine	11 weeks

*****Patient 10 had a double prescription for morphine: 7.5mg + 20 mg

Table A.3

Additional drugs and time since first cannabinoids intake

Study Id	ATC treatment - deprescription	Time
2	fentanyl	40 weeks
	oxazepam	20 weeks
9	morphine	12 weeks
12	oxazepam	40 weeks
16	melitracen et flupentixol	14 weeks
17	mirtazapine	38 weeks

Supplement B

Table B.1
Metabolic ratios for CYP 1A2, 2B6, 2C9, 2C19, 2D6, 3A4

		Min	Q1	Median	Q3	Max	Mean	±SD
CYP1A2	1st	0.16	0.19	0.25	0.29	0.59	0.26	±0.11
	2nd	0.12	0.15	0.17	0.22	0.46	0.20	±0.09
CYP2B6	1st	0.80	1.53	2.11	2.99	3.99	2.26	±1.00
	2nd	1.21	2.20	3.38	3.82	5.79	3.14	±1.30
CYP2C9	1st	0.06	0.06	0.08	0.09	0.12	0.08	±0.02
	2nd	0.04	0.05	0.06	0.06	0.10	0.06	±0.02
CYP2C19	1st	0.22	0.29	0.38	0.57	1.47	0.55	±0.38
	2nd	0.10	0.25	0.34	0.43	1.91	0.44	±0.44
CYP2D6	1st	0.02	1.00	1.61	2.62	3.41	1.72	±1.06
	2nd	0.02	0.96	1.66	2.66	4.87	1.83	±1.35
CYP3A4	1st	0.51	0.63	0.81	1.08	1.55	0.89	±0.32
	2nd	0.31	0.73	0.84	1.00	1.44	0.85	±0.27

Metabolic ratio= metabolite concentration/substrate concentration
1st= first blood sampling; 2nd= second blood sampling
For full patient results confront Table B.2

Table B.2

Per patient metabolic ratios after micro cocktail intake, first (1st) and second (2nd) blood sampling, for CYP 1A2, 2B6, 2C9, 2C19, 2D6, 3A4

**Subj Id**	**CYP1A2**		**CYP2B6**		**CYP2C9**		**CYP2C19**		**CYP2D6**		**CYP3A**	
	**1st**	**2nd**	**1st**	**2nd**	**1st**	**2nd**	**1st**	**2nd**	**1st**	**2nd**	**1st**	**2nd**
1	0.29	0.20	0.90	2.46	0.08	0.05	0.29	0.44	0.02	0.02	0.82	0.56
2	0.27	0.18	0.80	3.91	0.08	0.07	1.08	0.49	0.81	0.75	0.80	0.31
5	0.30	0.14	2.11	4.72	0.08	0.10	0.56	0.36	1.88	1.83	0.51	0.92
6	0.16	0.14	3.31	3.42	0.12	0.05	1.47	0.46	2.82	1.15	1.10	0.74
7	0.18	0.16	2.86	3.51	0.08	0.08	0.58	0.33	3.41	2.63	1.02	0.87
8	0.25	0.17	1.26	1.23	0.06	0.04	0.23	0.10	1.48	1.22	1.43	1.44
9	0.33	0.29	2.52	1.21	0.10	0.05	0.37	0.41	0.03	0.03	1.11	0.68
10	0.28	0.23	2.06	3.35	0.09	0.06	1.08	1.91	2.26	3.40	1.55	1.16
11	0.18	0.17	1.83	3.54	0.05	0.06	0.28	0.30	1.61	1.62	1.26	0.88
12	0.19	0.12	3.63	5.79	0.06	0.06	0.29	0.26	1.59	1.70	0.59	0.82
13	0.59	0.46	3.04	2.87	0.09	0.06	0.40	0.38	1.51	0.89	0.55	0.73
14	0.24	0.23	3.99	4.06	0.08	0.06	0.47	0.22	0.85	2.67	0.74	0.79
15	0.20	0.18	1.50	2.11	0.06	0.06	0.37	0.21	3.04	4.87	0.87	1.03
19	0.17	0.15	2.12	1.82	0.06	0.04	0.28	0.24	1.64	2.88	0.60	1.02
17	0.43	/	3.09	/	0.06	/	0.69	/	2.52	/	0.71	/

Reference values for Geneva micro cocktail phenotyping PM=poor metabolizer; EM=normal metabolizer; UM=ultra metabolizer

	CYP1A2	CYP2B6	CYP2C9	CYP2C19	CYP2D6	CYP3A4
PM	0.03-0.204	0.062-0.13	≤0.03	0.08-0.28	0.03-0.07	0.15-0.29
EM	0.17-0.39	0.81-2.97	>0.04	0.3-1.22	0.62-4.3	0.32-0.82
UM	0.42-0.70	4.9-12.7	≥1.3	2.96-7.88		2.24-5.25

Table B.3 – THC, metabolites, and CBD plasma concentrations
THC, 11-OH-THC, THC-COOH and CBD plasma concentration mean values (± SD) and corresponding 24 hours THC or CBD dosages

Number of Patients/ Analysis	THC admin mg/24h	THC µg/L Mean (±SD)	11-OH-THC µg/L Mean (±SD)	THC-COOH µg/L Mean (±SD)	CBD admin mg/24h	CBD µg/L Mean (±SD)
**2/2**	**0**	Not detectable	Not detectable	Not detectable	**0**	Not detectable
**3/5** of which 1/ 2 extra CBD	**9.6**	1 (±0.0)	1 (±0.0)	22.6 (±7.4)	**19.2**	1 (±0.0) 14.9(±8.7) with extra CBD
**3/4**	**12**	1.1 (±0.1)	1.2 (±0.2)	32.2 (±10.6)	**24**	2.2 (±1.0)
**1/2**	**13.2**	1.4 (±0.6)	1.4 (±0.5)	62.5 (±13.4)	**26.4**	1.9 (±1.3)
**8/9**	**14.4**	1.8(±1.1)	2.8(±2.7)	74.4 (±19.2)	**28.8**	2.9 (±1.3)
**4/5**	**15.6**	1 (±0.0)	1.6 (±0.3)	48.0 (±33.2)	**31.2**	2.1 (±0.8)
**1/1**	**16.8**	1	1.6	86.0	**33.6**	2.8
**1/2**	**18**	2.9 (±2.1)	2.6 (±1.6)	61.0 (±35.4)	**36**	3.8 (±3.5)
**1/1**	**19.2**	1	1.6	84.0	**38.4**	3.5

- Values of 3 samples were excluded since the THC/CBD treatment was administered 1 hour before blood sampling by mistake
- Values of 1 sample (THC 8.4mg/24H – CBD 16.8 mg/24 hours) were missing.
- Values for 1 patient were collected only at first sampling
- At both samplings, 1 patient took extra CBD as a co-medication for treating epilepsy on physician prescription.
- Values equal or inferior to 1 were calculated as 1 for statistical purposes.

THC, 11-OH-THC and CBD: **LOD:** 0.5µg/L; **LLOQ:** 1.0 µg/L
THC-COOH: **LOD:** 1.0µg/L; **LLOQ:** 2.5 µg/L
LOD = limit of detection; LLOQ =lower limit of quantitation

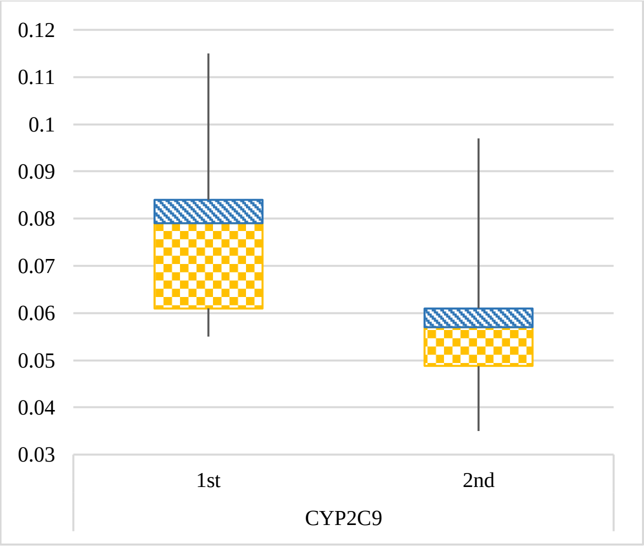

Figure B.1
CY2C9 enzymatic activity.
Box Plot representing the metabolic ratios at 1st and 2nd blood sampling.
Geneva cocktail reference values: PM (poor metabolizer) <0.03; EM (normal metabolizer) >0.4; UM (ultra metabolizer) >1.3

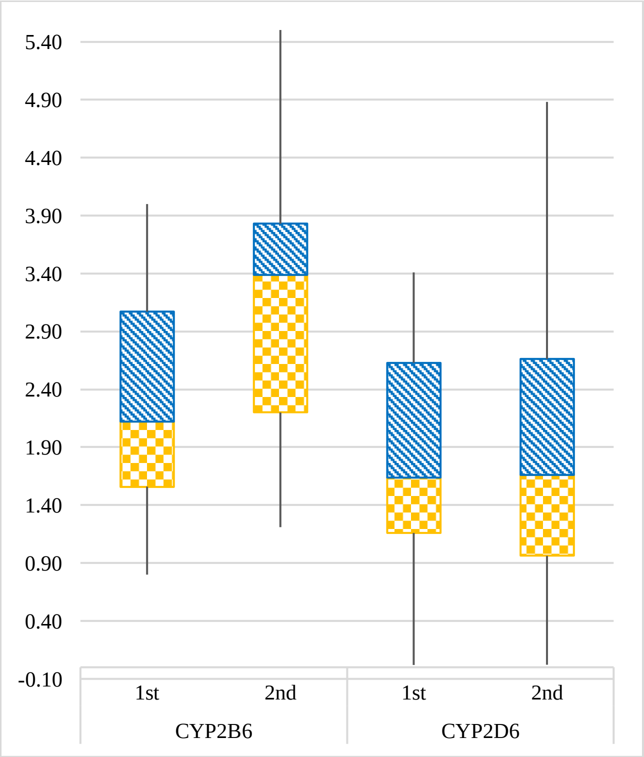

Figure B.2: CYP2B6, CYP2D6 enzymatic activities.
Box Plot representing the metabolic ratios at 1st and 2nd blood sampling.
Geneva cocktail reference values (-SD to +SD): CYP2B6= PM (poor metabolizer) 0.062-0.13; EM (normal metabolizer) 0.81-2.97; UM (ultra metabolizer) 4.9-12.7. CYP2D6= PM (poor metabolizer) 0.03-0.07; EM (normal metabolizer) 0.62
